# The endocrine pathway during whole body vibration modulates the immune system and the osteogenesis in postmenopausal women with osteoporosis

**DOI:** 10.3389/fendo.2026.1776499

**Published:** 2026-05-15

**Authors:** Vasiliy Pyatin, Olga Maslova

**Affiliations:** 1 Neurointerfaces and Neurotechnologies Laboratory, Neurosciences Research Institute, Samara State Medical University, Samara, Russia; 2 Science Department, Eurasian Technological University, Almaty, Kazakhstan

**Keywords:** endocrine pathway, immune system, osteoimmunology, osteoporosis, post-menopausal women, whole body vibration (WBV)

## Abstract

**Background:**

Osteoimmunology is an interdisciplinary field that investigates the interaction between the immune and skeletal systems in the context of immune regulation of bone remodeling. However, to date, osteoimmunology has not been linked to the broader systemic regulation of osteogenesis during physical exercise. Whole-body vibration (WBV) exercise initiates systemic sensory, endocrine, immune, and osteogenic responses. However, there is a gap in the understanding of the mechanisms underlying cross-system integration in WBV.

**Objectives:**

Our working hypothesis is that brain-immune modulation of osteogenesis in postmenopausal women with osteoporosis under the WBV conditions is regulated by the integrative proprioceptive endocrine pathway.

**Methods and objects:**

The postmenopausal women 65–67 years old with osteoporosis participated in the study. Methods are WBV, DEXA, laboratory methods for analyzing pro-osteogenic mediators, cellular and humoral immunity. Statistics is performed by StatPlus package; statistically significant changes in mean values ​​at p < 0.05.

**Results:**

In postmenopausal women with osteoporosis under the 6-months WBV conditions the bone mineral density increased by 1.56% (p <0.05) and the peripheral blood lymphocyte subpopulations and serum humoral immunity was changed.

**Conclusion:**

The data obtained allow us, for the first time, to propose a hypothesis regarding brain-immune interaction in the regulation of osteogenesis in postmenopausal women with osteoporosis, the interface for which in cross-system interaction is the integrative proprioceptive endocrine pathway, which generally links the brain, the immune system and the bone remodeling during longitudinal WBV.

## 1 Introduction

Osteoimmunology is an interdisciplinary field that investigates the role of immune cells and factors in maintaining bone homeostasis (1). It is believed that physical activity modulates bone metabolism through biomechanical loading, muscle tension on the bone, and via the immune system (2). However, there is a knowledge gap in the literature when it comes to physical activity—and particularly activities such as WBV—and osteoimmunology (3).

The systemic osteogenic mechanisms underlying the effects of WBV have not been the subject of an inter-system analysis, and this gap in the integrated knowledge persists to this day. For the first time, at the experimental and hypothetical levels, we substantiate the role of the interaction between the brain and the immune system in osteogenesis th\gh the modulating effects of the integrative proprioceptive system on the endocrine pathway. This framework represents the foundational hypothesis of our study and a promising paradigm for the development of rehabilitation strategies in the elderly adults, especially in postmenopausal women with osteoporosis and age-related involution of endocrine regulatory functions. Therefore, the aim of our study is to experimentally confirm the working hypothesis that in elderly postmenopausal women with osteoporosis the integrative proprioceptive system during WBV activates osteogenesis by recruiting the immune system. The brain-hormone-immune framework forms the basis of osteogenic regulation.

WBV, in its rehabilitative application, has demonstrated proven potential for enhancing and restoring musculoskeletal function (4, 5), as well as for the integrative regulation of CNS functions through the proprioceptive system (6).

Both bone and muscle tissues of the human body serve as the targets for the effects of the acceleration and WBV acceleration frequency. In bone tissue, the targets of WBV for bone remodeling are osteoblasts and osteocytes, which originate from mesenchymal stem cells, and osteoclasts, which derive from hematopoietic stem cells. Osteoblasts form bone tissue, osteoclasts resorb and even destroy it, while osteocytes control the activity of osteoclasts and osteoblasts, ensuring the structural integrity and function of the bone (7). Disruption of the balance of intercellular interactions among osteoblasts, osteocytes, and osteoclasts is a key factor leading to osteopenia and osteoporosis. In particular, osteoporosis can be caused by increased bone resorption by an active pool of mature osteoclasts, with bone fracture being one of its most serious health consequences (8).

Osteoblasts constitute the main cellular population of bone tissue and are the terminally differentiated products of mesenchymal stem cells. They perform three main functions: cellular regulation of bone formation; regulation of osteoclast differentiation through the expression of macrophage colony-stimulating factor (M-CSF) and receptor activator of nuclear factor κβ ligand (RANKL) (9). Osteoblasts modulate the osteoclasts functions through the secretion of proteins: osteoprotegerin (OPG), by binding RANKL, prevents the interaction of RANKL and RANK; osteoblast calcineurin/NFAT, through chemoattractant signaling mechanisms (CL8, CCL6, CCL12), regulates osteoclast differentiation and recruit of osteoclast precursors to the bone (10–12). Osteoblasts are involved in the microenvironment of HSC and are closely associated with its function (13). Mature osteoblasts secrete several extracellular proteins essential for bone formation, including type I collagen (COL1), osteocalcin (OCN), bone sialoprotein II (BSP II), and osteopontin (OPN) (14–16).

Following bone tissue formation, osteoblasts can undergo one of three states: 60–80% of osteoblasts undergo apoptosis, transform into bone tissue cells, or become osteocytes (17, 18). Regulation of osteoblastogenesis and osteoclastogenesis is a complex process involving multiple canonical and non-canonical signaling pathways (17, 19). The canonical pathways influencing osteoblast differentiation include Wnt, TGF-β, Hedgehog, and FGF signaling pathways (20–25). Other signaling pathways such as Notch, Hippo, and NF-κB also contribute to osteoblast differentiation (7, 26–28).

Osteoclasts maintain bone tissue homeostasis by possessing a pronounced capacity to resorb bone. Osteoclast precursors are directed to the bone resorption surface from the bone marrow and bloodstream via sphingosine-1-phosphate signaling. Subsequently, they fuse into multinucleated osteoclasts, which are associated with the presence of RANKL and macrophage colony-stimulating factor (M-CSF) (29). Upon stimulation, osteoclasts activate a broad spectrum of signaling cascades, including canonical and non-canonical NF-κB pathways, MAPK, calcium signaling, and PI3K/AKT pathways (30). Osteoclast differentiation and bone resorption are controlled by signaling pathways, whose imbalance serves as a key mechanism underlying metabolic bone disorders (29).

The main regulator of osteoclast differentiation is the RANKL/RANK/OPG axis, in which RANKL-RANK binding initiates subsequent signaling cascades, while OPG acts as a critical decoy receptor to maintain bone tissue homeostasis. The spatiotemporal dynamics of RANKL expression occur in interaction with immune cells, being an important mechanism of interaction between bone tissue and immune system (31). The NF-κB signaling pathway, particularly its canonical branch, serves as a central link integrating inflammatory signals (e.g., TNF-α, IL-1β) with osteoclast activation. The MAPK/ERK signaling pathway further enhances osteoclast activity through CREB-mediated transcriptional activation of c-Fos and NFATc1, highlighting its synergistic role with RANKL and NF-κB signaling pathways (32).

The RANKL/RANK/OPG signaling axis, being a key regulator of bone tissue homeostasis, plays a crucial role in physiological and pathological bone remodeling, dynamically balancing osteoclast differentiation and bone resorption. Furthermore, multinucleated osteoclasts (mOCs) exhibit polarity and adhere to the bone surface, forming a resorption lacuna by secreting lysosomal proteases, including MMPs, CTSK, and TRAP, into which hydrochloric acid is actively secreted, leading to the dissolution of the bone mineral hydroxyapatite. In addition to RANK/RANKL and M-CSF, other factors have been shown to be involved in osteoclastogenesis, such as Wnt, FGF, and the transforming growth factor beta (TGF-β) pathway (33). TNF-α and IL-1β synergistically enhance the RANKL expression in osteoblasts and fibroblasts, thereby accelerating osteoclast differentiation and bone resorption through paracrine signaling (29, 34).

Osteocytes are a major component of the skeleton that maintains the bone tissue environment and participates in the regulation of osteoblasts and osteoclasts activity during bone remodeling (35). Osteocytes are embedded in bone tissue, forming a complex network within the bone structure and maintaining close contact with the other bone cells and bloodstream. Osteocytes secrete RANKL to induce osteoclast differentiation and OPG to inhibit osteoclast formation (36). In addition, osteocytes produce FGF-23, BMPs, and sclerostin (SOST), regulating osteoblasts activity. These functions largely depend on endocrine, paracrine, and autocrine mechanisms regulating bone tissue homeostasis (18).

Osteocytes act as mechanosensors of bone tissue, and by perceiving mechanical signals, they convert them into signaling processes of specific ion channels (37). These cells are stimulated by various mechanical forces in the bone, gravitational loading, and daily physical activities, leading to changes in interstitial fluid flow within Haversian canals and matrix deformation at the cellular level in the bone. The cellular response of osteocytes to mechanical stimulation is crucial for both viability and the regulatory role in balanced bone homeostasis (38).

Recent studies have identified the mechanosensitive ion channels Piezo1 as essential force sensors required for bone development and for regulating osteoblast differentiation and function (39–41). *In vivo* studies have demonstrated the essential role of Piezo1 in the osteoblastic lineage, albeit with varying phenotypes (42). Patients with osteoporosis exhibit reduced Piezo1 protein levels, and conditional knockout of Piezo1 in osteoblast cell lines impairs osteogenesis by suppressing RUNX2, COL1, and OCN expression (40). Activation of Piezo1 in osteocytes triggers a downstream cascade, including the Wnt/β-catenin signaling pathway, which enhances osteogenesis and suppresses bone resorption, as evidenced by impaired bone formation and increased osteoclast activity in conditional knockout models (43). As mechanosensors, osteocytes detect fluid shear stress and matrix deformation through dendritic processes and primary cilia, converting them into biochemical signals that modulate bone remodeling (38, 44). The mechanosensory ion channel Piezo1, which facilitates the exchange of ions between the cell and the extracellular environment, leads to the activation of voltage-sensitive calcium channels (44).

Piezo1 is expressed in both osteoblasts and osteocytes, thereby regulating mechanical load-dependent bone formation (40, 41). Deficiency of mechanosensitive Piezo1 channels results in explicited hyperactivity of osteoclasts relative to osteoblastic activity (45). Lacked mechanosensitivity to mechanical loads of the bone dysregulates the osteoblast-osteoclast interaction, leading to bone loss (42, 46). Mechanical stress affects the bone microenvironment and metabolic activity (41). Moreover, osteocytes also appear to use the Wnt/β-catenin pathway to transmit mechanical load signals to the cells (41, 47). These studies confirm that the bone microenvironment and non-canonical Wnt signaling pathways are actively involved in mechanotransduction (47). Bone morphogenetic protein-2 (BMP-2), a member of the transforming growth factor β (TGFβ) superfamily, is involved in mechanotransduction. BMP-2 can also act in coordination with other mechanosensitive signaling pathways, thus participating in the formation of bone, cartilage, and joints (42).

During WBV exposure, deformations resulting from mechanical stress within the complex bone tissue, along with their direct consequences, such as changes in intralacunar pressure and fluid flow within the bone, serve as the targets for WBV stimulation and as a factor for increasing bone mass (48). It is well recognized that aging is accompanied by the processes of functional involution of physiological systems, including the musculoskeletal and immune systems, and a decline in the bidirectional interactions between these systems (49). However, prior studies have shown that strength training used in clinical settings to improve bone quality promotes the increased expression of genes associated with the Wnt signaling pathway regulating osteogenic homeostasis (50).

### 1.1 Endocrine pathway imbalance during post-menopause

In postmenopausal women, a marked decline in ovarian estrogen (E2) production contributes to impaired bone metabolism, affects key signaling pathways associated with bone metabolism, and regulates immune system function (51). Normally, estrogen 17β modulates the expression of several osteoblast-derived factors. For example, it increases the osteoprotegerin (OPG) level and decreases the level of receptor activator of nuclear ligand κB (RANKL) and tumor necrosis factor (TNF)-α levels, which leads to suppression of bone resorption (52). The OPG-RANKL-RANK signaling pathway is the primary pathway regulating bone resorption. The balance among OPG, RANKL, and RANK is critical for maintaining bone mineral density (BMD) (53).

Estrogens maintain bone homeostasis by dually regulation OPG and RANKL expression (52). In this case, osteoclast production is inhibited, and BMD is maintained through increased OPG expression and decreased RANKL expression. In case of estrogen deficiency, the RANKL expression increases, OPG expression decreases, and the OPG/RANKL ratio reduces (54). Bone resorption activity is regulated by the OPG/RANKL ratio. When the OPG/RANKL ratio is high, bone resorption is suppressed; when the OPG/RANKL ratio is low, osteoclast activity is enhanced and bone resorption increases (55). Excess RANKL binds to RANK on the osteoclast surface, stimulating osteoclast differentiation and activity, and therefore increasing bone resorption (31). Bone tissue formation cannot fully compensate for the increased bone resorption, ultimately leading to decreased BMD and postmenopausal osteoporosis (PMOP).

The canonical Wnt/β-catenin signaling pathway plays a pivotal role in maintaining bone metabolic homeostasis (22). Following menopause, decreased estrogen levels lead to increased expression of Dkk1 and sclerotin (SOST), which inhibit the Wnt signaling pathway and impair bone metabolism (21). Inhibition of the Wnt/β-catenin signaling pathway results in decreased OPG expression and increased RANKL expression, disrupting the OPG-RANKL-RANK pathway balance and enhancing bone resorption (21). As estrogen levels decline, the interaction between these two signaling pathways creates a vicious cycle that further worsens postmenopausal osteoporosis.

Thus, the OPG-RANKL-RANK and Wnt/β-catenin are two important signaling pathways influencing PMOP. The OPG-RANKL-RANK pathway is primarily associated with osteoclast inhibition, and the Wnt/β-catenin pathway regulates the osteoclast production. Estrogen deficiency promotes osteoporosis by increasing RANKL expression and decreasing OPG expression. This mechanism is particularly pronounced in postmenopausal women, as decreased estrogen levels lead to an imbalance in bone remodeling, increased bone resorption, and decreased bone formation. Studies using bone marrow transplantation models and chimeric mouse models have shown that estrogen directly suppresses RANKL transcription in bone lining cells via estrogen receptor α (Erα), thereby inhibiting osteoclast activity. RANKL forms an “*in situ* signaling pool” on the bone surface, regulating the differentiation of adjacent osteoclast precursors (54). However, estrogen affects not only bone cells, but also postmenopausal osteoporosis, as this is a condition that affects the entire body. In particular, this concerns bone tissue metabolism. Although the role of bone metabolism, mitochondrial function and oxidative stress are known to contribute to postmenopausal osteoporosis under estrogen deficiency (56), these metabolic aspects were beyond the scope of our current working hypothesis.

Estrogens control the immune system activity through interaction with membrane receptors of the innate immune system (neutrophils, macrophages/monocytes, natural killer cells, dendritic cells) and the adaptive immune system (T and B cells) cells (57). Estrogens are also known to interact with various immune cells, leading to a chronic, low-grade proinflammatory phenotype in estrogen deficiency. This subsequently leads to increased osteoclast production and activity (12).

The interaction between the bone cells and immune system occurs through cellular and molecular processes within the bone marrow microenvironment (58). Moreover, osteoclasts, as a cells of the monocytic lineage, have a wide range of phenotypic and functional heterogeneity associated with anti- and pro-inflammatory effects and antigen presentation depending on the environment (59). Immune cells engage with bone cells at multiple regulatory levels. Therefore, it is suggested that postmenopausal bone loss is partially mediated by interaction between immune cells and bone metabolism (60).

### 1.2 Whole body vibration for the restoration of bone mineral density

Mechanical loading plays a crucial role in maintaining the balance between bone formation and bone resorption (42). This osteogenic stimulus depends on multiple parameters of the deformation environment, such as the number, rate, peak magnitude, direction and distribution of deformation. As we noted previously, osteocytes can be stimulated by various mechanical forces in the bone generated by gravity and daily physical activities, which induce changes in interstitial fluid flow and matrix deformation at the cellular level in the bone. The cellular response of osteocytes to mechanical stimulation is essential for viability and for its regulatory role in balanced bone homeostasis (38). These mechanical stimuli are detected by osteocytes, which are the key mechanosensitive cells of the bone involved in bone tissue restoration and remodeling, along with osteoblasts, osteoclasts, chondrocytes, and mesenchymal stem cells (61).

Recent studies have demonstrated that vibration therapy activates osteoblast differentiation pathways (e.g., Wnt/β-catenin signaling pathway), increases the expression of osteogenic markers such as Runx2, BMP-2, and OPG, and suppresses osteoclast activity reducing the RANKL/OPG ratio in the bidirectional regulation of bone metabolic balance (62). Vibration therapy significantly increases the expression of Wnt-related proteins, while simultaneously decreasing the receptor activator of nuclear factor kappa-B (RANKL)/OPG ratio, thereby inhibiting osteoclast proliferation and promoting dynamic balance between bone resorption and formation. This mechanism theoretically confirms the role of vibration therapy in regulating bone metabolism (63, 64). In an experimental study in laboratory ovariectomized animals, it was shown that the effect of WBV on muscle mass and strength was greater in the presence of estrogen than with WBV alone. The combination of WBV with E2 significantly increases the Akt and mTOR activity and decreases the FoxO1 activity (65). These signaling pathways are key mechanisms through which physical exercises exert its effects on various biological systems, including muscle power and strength, by influencing cellular homeostasis, muscle adaptation, metabolic regulation, and neuroprotection (66). Numerous studies have documented the effects of WBV on the human endocrine system. Changes in the concentrations of hormonal and non-hormonal biomarkers following WBV are suggested to be due to neuroendocrine interactions (67, 68).

Within the framework of our working hypothesis, in this publication we highlight the role of the integrative function of the proprioceptive system in the cellular mechanisms of bone tissue remodeling and the immune system responses during WBV. We also substantiate the hypothesis that the endocrine pathway is the interface between the brain and the immune system on the one hand, and between brain and osteogenesis, on other hand. This hypothesis gains particular relevance given the limited number of recent studies investigating the effects of WBV on brain function and the immune system of older adults (69).

## 2 Objects and methods

### 2.1 Patients

The study involved elderly postmenopausal women diagnosed with femoral osteoporosis. In accordance with the study design, an experimental group (20 women, aged 65 ± 4 years) was formed using a non-randomized method in accordance with the inclusion criteria, and this group took part in a 6-month WBV program. The women in the experimental group did not take calcium or vitamin D supplements and did not undergo antiresorptive therapy. Consequently, women with features of osteoporosis were not included in the experimental group, but they met the exclusion criteria for the experimental group. Two control groups took part in the study. One control group (CG1 of elderly postmenopausal women diagnosed with femoral osteoporosis, n=20, 66 ± 4 years old) did not participate in the WBV program but took calcium (1000 mg/day) and vitamin D (800 IU/day) supplements during the study period. This control group participated in a comparative study with the experimental group, focusing on the markers of bone remodeling. Another control group (CG2 of elderly postmenopausal women diagnosed with femoral osteoporosis, n=19, aged 67 ± 5 years) did not take any medication during the study period. This group took part in a comparative study of peripheral blood leukocyte counts and cellular and humoral immune factors. No participants dropped out of the study or were excluded during the six-month program.

The study protocol was approved by the Ethics Committee of Samara State Medical University and included three stages (“Before”, after month 3 and after month 6) of blood sampling and detecting the BMD of the femur. The study design is presented in **Table 1**.

**Table 1:** Study design of the 6-months Whole Body Vibration program for women in the experimental group and control groups.

Start (0 months)	Experimental group (n=20)			
	Power Plate sessions	After month 3	Power Plate sessions	After month 6
Osteodensitometry Immune assay Blood simple	3 months, 3 time per week	Osteodensitometry	3 months, 3 time per week	Osteodensitometry
		Immune assay		Immune assay
		Blood simple		Blood simple
	Control group 1 (n=20)			
	Not Power Plate sessions	Osteodensitometry	Not Power Plate sessions	Osteodensitometry
		Blood simple		Blood simple
	Control group 2 (n=19)			
	3 months, 3 time per week	Immune assay	3 months, 3 time per week	Immune assay

### 2.2 Inclusion criteria

1) women aged 60 to 75 years; 2) no previous Power Plate training experience; 3) informed consent to participate in the study; 4) diagnosed osteoporosis; 5) the absence of absolute contraindications to WBV intervention such as oncological and immunoproliferative diseases, autoimmune diseases, acute inflammatory diseases, the presence of a pacemaker and a spinal cord stimulator, skin diseases, osteoporosis with a level 4 risk of bone fracture, spinal hernias, discopathy, joint endoprosthesis, cholelithiasis, urolithiasis, migraine, epilepsy, type 1 diabetes mellitus, severe cardiovascular diseases, stage III arterial hypertension. Conversely, these absolute contraindications to WBV were considered as *exclusion criteria*.

### 2.3 Osteodensitometry

Non-invasive bone mass assessment was performed using dual-energy X-ray absorptiometry to measure BMD in the proximal femur. The study of women was conducted in the standard position with an anteroposterior scan of the proximal right femur. BMD was calculated in grams per cm². The method had an accuracy of 4.5% and a reproducibility of 1–2%. The radiation dose per examination was approximately 1–3 mR, depending on the beam geometry. The BMD of the femur in women was determined using dual-energy X-ray absorptiometry (DXA) with a “Norland XR-46” osteodensitometer (USA). Osteoporosis was diagnosed using standard procedures, in accordance with the recommendations of the National Osteoporosis Foundation (USA).

### 2.4 Laboratory methods

Blood samples from the women were collected in the morning from the cubital vein, 24 hours after a WBV session at the stages “Before”, after month 3, and after month 6. Venous blood samples were collected by a certified medical professional. A complete blood count (CBC) was performed using a Cell-Dyn 3700 analyzer (USA) with Abbott reagent kits (USA).

#### 2.4.1 Biochemical and immune tests

Immunoglobulins A, M and G in blood serum were detected by an automatic biochemical analyzer “Hitachi-902” (Japan) with reagents “Roche-Diagnostivs” (Switzerland). The concentrations of immunoglobulins A, M and G in blood serum were determined using an immunoturbidimetric method based on the reaction between antibodies and immunoglobulins A, M and G. Total calcium and phosphorus levels were determined by a “Cobas Integra” analyzer (Switzerland) and test kits. Plasma osteocalcin levels were measured using radioimmunoassay (the “N-MID Osteocalcin” test kit and the “Elecsys” analyzer), whilst β-CrossLaps were measured using a cross-linked immunoassay (the “Elecsys β-CrossLaps” test kit and “Elecsys” analyzer). The concentration of proinflammatory cytokines in the blood serum (TNFα, IL8) was determined using an enzyme-linked immunosorbent assay (ELISA) system comprising the “Uniplan” reader (Picon, Russia), the “Elmi Sky Line” shaker and the “Uniplan” spectrophotometer (Picon, Russia). The test systems “TNF∝-IFA-Best” and “Interleukin-8-IFA-Best” reagent kits (Vector-Best, Russia) were used. The method is based on a “sandwich” option of the solid-phase enzyme-linked immunosorbent assay, involving the highly specific binding of cytokine antibodies to polystyrene, which is capable of extracting cytokines from blood plasma.

#### 2.4.2 Immunophenotyping of lymphocytes

Lymphocyte immunophenotyping was performed using a multi-parameter, two-color immunofluorescence assay stained with monoclonal antibodies (LLC Sorbent, Russia) on the flow cytometer “Facs Calibr” (Becton Dickinson, USA). The flow cytometry method involved detecting the light scattering of a laser beam as cells passed through it in a liquid stream: light scattering was measured at small angles (from 1^0^ to 10^0^), as well as at 90^0^, and fluorescence intensity was measured across four channels. Monoclonal antibodies labelled with the fluorochromes FITC and PE (CD3, CD4, CD8, CD16, CD20, CD25, HLA-DR) were used to stain the cells. Erythrocytes were removed using lysis solutions OptiLyse C, OptiLysB, ImmunoPrep, Whole Blood Lysing Reagent (Beckman Coulter, USA). Mathematical processing of cytometric data was analyzed by EXPO-32 and CXPv.2.2 software from “Beckman Coutler” (USA). Approximately 10^4^ cells were analyzed in each sample.

### 2.5 Whole body vibration procedure and program

The 6-months WBV program on the Power Plate Pro5 (Power Plate North America, Chicago, IL, USA) consisted of three workouts per week with a 48-hour rest interval. The progression of static WBV strength exercises involved increasing frequency - from 30 Hz (month 1) to 40 Hz (month 6), and exercise duration - from 30 sec (month 1) to 45 sec (month 6). Rest time between the strength exercises was 45–60 sec. The platform motion amplitude was constant (2 mm). The duration of each session was 20–25 min. The program of each session included 5 warm-up exercises at a constant WBV mode (30 sec, 30 Hz); 5 strength exercises for the leg and arm muscles in a progression mode; 5 recovery exercises (massage and relaxation of the leg muscles) at a constant WBV mode (40 Hz, 60 sec). **Table 1** presents the design of the WBV program. The protocol for the WBV program was strictly adhered to throughout the study. All WBV sessions on the Power Plate platform were carried out individually and supervised by a certified Power Plate specialist.

### 2.6 Statistics

Statistical analysis was performed using StatPlus package containing a preliminary analysis of the distribution of all recorded parameters. Parametric method for dependent and independent groups, and non-parametric methods (Mann-Whitney and Kolmogorov-Smirnov tests) were used. The difference between the mean values of the characteristic under study across the groups of subjects was determined using a parametric method. The results were presented as mean values ​​and their standard errors. The reliability of the measurements was assessed using the two-sample t-test. Changes in mean values were statistically significant ​​ at p < 0.05. Non-parametric methods were used for characteristics difference from the norm. Quantitative or ordinal variables were used when comparing two groups by the Mann-Whitney test. The hypothesis of equality of mean ranks was tested. Using the Kolmogorov–Smirnov two-sample test, we tested the hypothesis that the groups of characteristics under investigation were drawn from the same population.

## 3 Results

### 3.1 Bone remodeling

The 6-months WBV program did not affect the serum phosphorus and calcium levels in the EG and CG. **Table 2** shows the levels of BMD, Osteocalcin and β-CrossLaps during the WBV 6-months program. Consequently, the increase in the concentration of the multifunctional hormone osteocalcin following a 6-month WBV program in the EG is a marker of bone turnover, and it also functions as a versatile endocrine mediator at the interface of bone physiology and the systemic metabolic regulation (70). The osteocalcin difference between the EG and CG groups was *p* < 0.01. Under these conditions, the serum levels of the bone resorption marker β-CrossLaps in EG and CG did not differ between the three stages of the WBV program. As is well known in the case of osteoporosis, bone resorption increases while the degradation of type I collagen intensifies, leading to elevated blood levels of b-CTX, a collagen degradation product reflecting osteoclastic activity (71).

**Table 2:** Particularity of the BMD, Osteocalcin and β-CrossLaps during the WBV 6-months program.

Parameter	Whole Body Vibration		
	Before	3 months	6 months
BMD (g/cm2)	0.7367 ± 0.0134	–	0.7482 ± 0.0137*
Osteocalcin (ng/ml)	21.80 ± 2.27	30.76 ± 1.95*	32.70 ± 2.61*
β-CrossLaps (ng/ml)	0.376 ± 0.06	–	0.418 ± 0.036

* p < 0.05 after month 3 or month 6 compared to the “Before” stage.

BMD of the femur in EG postmenopausal women after 6 months of the program increased by 1.56% (*p* < 0.05). In CG women, the BMD values measured at baseline and after 6 months of the study remained essentially unchanged (0.7262 + 0.015 g/cm^2^ and 0.7667 + 0.0071 g/cm^2^, respectively).

### 3.2 Innate and adaptive immunity during the 6-month WBV program

The body’s external barriers are a part of innate immunity (72). The response of cellular mechanisms of innate immunity is non-specific and universal, and many cells, including phagocytes (macrophages and neutrophils), dendritic cells, mast cells, basophils, eosinophils, natural killer (NK) cells, and innate lymphoid cells, participate in the innate immune response. Neutrophils and macrophages are the two main types of phagocytic cells.

As shown in **Table 3**, changes in the number of cells involved in innate immunity occurred differently throughout the study period. After 3 months, WBV caused a decrease in the number of neutrophils in the leukocyte formula, while their absolute number per one ml of blood increased (p<0.05). At the same time, both the percentage of monocytes in the leukocyte formula and their number in one ml of peripheral blood decreased.

**Table 3:** Changes of blood cells count during the 6-months of the Whole Body Vibration program.

Blood cells	Whole Body Vibration		
	Before	3 months	6 months
Neutrophils,%	53.98 ± 2.06	53.13 ± 1.76	49.71 ± 2.94*
Cells/ml	2967 ± 238	2751 ± 246	2442 ± 298*
Lymphocytes,%	35.01 ± 2.01	36.65 ± 1.43	40.3 ± 2.25*
Cells/ml	1862 ± 136	1843 ± 124	1885 ± 120
Monocytes, %	7.21 + 0.64	6.37 + 0.49	6.17 + 0.61*
Cells/ml	379 + 38	323 + 30*	294 + 32*
Eosinophils	2.78 + 0.38	2.85 + 0.44	2.97 + 0.49
Cells/ml	144 + 22	143 + 22	130 + 15
Basophils	1.02 + 0.09	0.98 + 0.08	1.13 + 0.18
Cells/ml	53 + 5	69 + 17	52 + 7

* p < 0.05 after month 3 or month 6 compared to the baseline

According to **Table 3**, the percentage of lymphocytes in the leukocyte formula increased in EG women after 3 months of the WBV program. Flow cytometry data on the lymphocyte population composition influenced by the WBV factors were identified after three months of participation of EG postmenopausal women with osteoporosis in the WBV program.

According to **Table 4**, it can be stated that in EG women by the month 3 of the WBV program, immune mechanisms (CD3+CD8+) are activated in, confirming by an increase in serum concentrations of proinflammatory cytokines. At the same time, a relative decrease was observed in CD3+CD4+ lymphocytes and CD3+CD8+ blood cells. After 6 months of the WBV program, in EG women, the serum levels of proinflammatory cytokines (IL-8, NFTα), immunoglobulins (A, M, G) and the subpopulation composition of blood lymphocytes did not differ from those recorded before participation in the 6-months WBV program.

**Table 4:** Particularity of the lymphocytes subpopulation during the 6-months Whole Body Vibration program.

Lymphocytes subpopulations	Whole Body Vibration		
	Before	3 months	6 months
CD3+, %	51.16 + 1.38	73.44 ± 1.27*	70.62 + 1.26**
CD3+, cells/ml	1336 + 117	1360 ± 99	1329 ± 85*
CD3+CD4+, %	44.27 + 1.65	42.67 ± 1.6*	43.3 + 1.81*
CD3+CD4+, cells/ml	825 + 59	776 + 51	806 + 47
CD3+CD8+, %	23.02 + 1.91	26.1 + 1.27*	21.85 + 1.94***
CD3+CD8+, cells/ml	427 + 64	488 + 46	422 + 52
CD3-CD8+, %	5.87 + 0.71	2.83 + 0.38**	6.18 + 0.83***
CD3-CD8+, cells/ml	100 + 12	48 + 8**	112 + 15***
CD3+CD16+, %	2.67 + 0.5	2.41 + 0.31	2.61 + 0.63
CD3+CD16+, cells/ml	53 + 12	48 + 8	49 + 12
CD3-CD16+, %	15/06 + 1.7	12.94 + 1.28*	13.01 + 1.71*
CD3-CD16+, cells/ml	264 + 33	235 + 28	250 + 44
CD3-CD20+, %	11.33 + 0.76	12.5 + 0.8	13.54 + 0.72*
CD3-CD20+, cells/ml	224 + 28	229 + 22	258 + 24
CD4+/CD8+	2.21 + 0.22	1.72 + 0.12*	2.22 + 0.27

*p < 0.05 after 3 months or 6 months compared to the baseline.

**p<0.01 after 3 months or 6 months compared to the baseline.

***p<0.05 after 6 months compared to 3 months of whole body vibration

The activation of the immune system in postmenopausal women with osteoporosis under the influence of the 6-months WBV program is evidenced by the levels of the late marker CD3+DR+ (**Table 5**).

**Table 5:** Particularity of the lymphocyte activation markers during the 6-months Whole Body Vibration program.

Lymphocytes	Whole Body Vibration		
	Before	3 months	6 months
Early activation markers			
CD3+CD25+, %	0.86 + 0.14	0.83 + 0.19	0.71 + 0.05
CD3+CD25+, cells/ml	16 + 3	15 ± 4	13 + 1
CD25+, %	0.97 + 0.15	0.92 + 0.2	0.79 + 0.06
CD25+, cells/ml	18 + 4	17 + 4	15 + 1
Late activation markers			
CD3+DR+, %	8.01 + 0938	4.83 + 0.7*	7.33 + 0.56**
CD3+DR+, Cells/ml	153 + 32	92 + 15*	136 + 16**

*p < 0.05 after 3 months or 6 months compared to the baseline.

**p<0.05 after 6 months compared to 3 months of whole body vibration

### 3.3 The humoral immunity by influence of the 6-months WBV program

Results of the three classes of immunoglobulins in serum (A, G, M) did not reveal significant changes under the influence of the 6-month WBV program (**Table 6**). The maximum IL-8 serum concentration was recorded after 3 months of participation in the WBV program (**Table 6**). These results significantly differed from the data obtained at the stage “Before” (+42.8%) and after 6 months of the WBV program (+20.76%). Significant changes in NFTα serum concentrations occurred at the 3rd month of the WBV program (**Table 6**). Moreover, after 6 months of the WBV program, NFTα levels significantly decreased below the values recorded at the “Before” stage. In CG women, no changes were observed across the three measurement points in any of the parameters of the complete blood count, as well as innate and adaptive immunity.

**Table 6:** Particularity immunoglobulins A, M, G and levels of cytokines in blood serum during the 6-months Whole Body Vibration program.

Immunoglobulins	Whole Body Vibration		
	Before	3 months	6 months
Ig A, g/l	2.607 + 0.1292	2.428 + 0.28	2.345 + 0.271
Ig G, g/l	8.82 + 0.528	8.364 + 0.75	8.069 + 0.683
Ig M, g/l	1.086 + 0.125	1.034 + 0.17	0.965 + 0.117
IL-8, pg/ml	6.988 + 1.673	9.977 + 1.481*	8.262 + 1.367
NFTα, pg/ml	2.1 + 0.547	4.216 + 1.1*	1.85 + 0.55**

*p < 0.05 after 3 months or 6 months compared to the baseline.

**p<0.05 after 6 months compared to 3 months of Whole Body Vibration

## 4 Discussion

**Figure 1:**
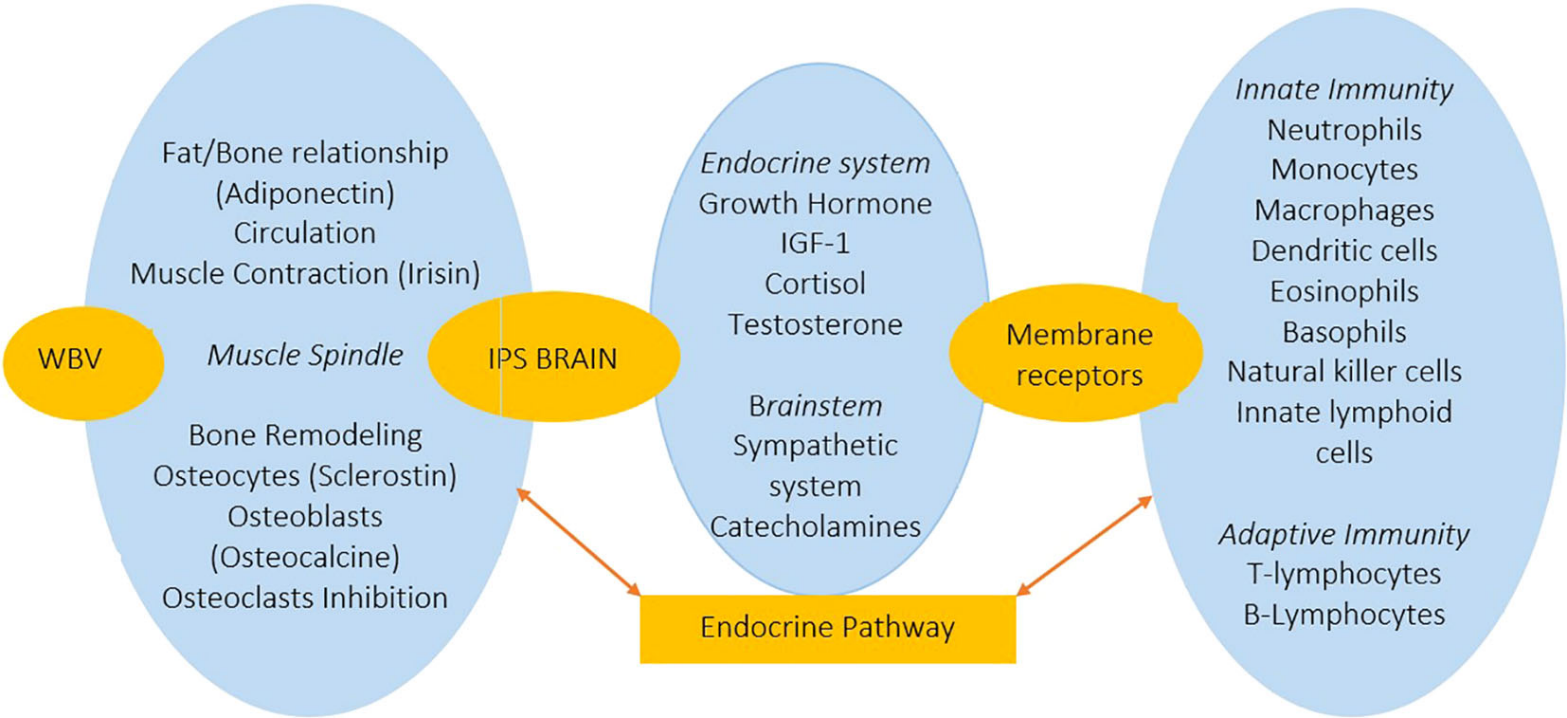
Schematic representation of the hypothesis about the role of primary bone mineral density remodeling in postmenopausal women with osteoporosis and the participation of the integrative proprioceptive system, which helps to eliminate the imbalance of hormone-immune regulation through the interaction of the brain-immune system via the endocrine pathway. The endocrine pathway modulates the bone remodeling through the osteoblasts, osteoclasts and osteocytes. WBV – whole body vibration; IPS – integrative proprioceptive system; IGF-1 - insulin growth factor.

According to our working hypothesis, the interaction between the integrative proprioceptive system and the musculoskeletal integration mechanisms during WBV eliminates the osteogenic and immune imbalance in postmenopausal women with osteoporosis. We argue that the integrative proprioceptive system during WBV modulates brain-immune interactions via an ascending endocrine pathway through the membrane receptors of innate and adaptive immune’s cells. This has been demonstrated in our study. Furthermore, according to the literature hormones secreted under the influence of WBV modulate osteogenesis through the descending endocrine pathway acting via the membrane receptors of osteoblasts and osteoclasts. The restoration of BMD observed in our study among postmenopausal women with osteoporosis during the initial three months of the WBV program indicates activation of the osteoblastic lineage in the bone. We postulate that in WBV, the osteoblastic bone line is modulated by the mechanosensory mechanism of osteocytes and hormones of the descending endocrine pathway (**Figure 1**). Research has shown that WBV is characterised by endocrine pathway response that includes increases in serum concentrations of growth hormone (73, 74), testosterone (67, 73), epinephrine and norepinephrine (75), as well as a decrease in cortisol levels (74). At the same time, receptors for these hormones are expressed on the membranes of cells of both the innate and adaptive immune systems.

During menopause, estradiol (E2) deficiency is closely associated with the changes in body composition, reduced muscle mass and strength in elderly women, and the development of osteoporosis. Our data demonstrate that WBV induces primary remodeling BMD in osteoporotic conditions and, despite estrogen-deficient menopause, modulates bone homeostasis, most likely through the dual regulation of OPG and RANKL expression. Normally, this occurs in the presence of estrogens in the blood plasma (8, 52).

As previously we wrote, the OPG/RANKL ratio regulates bone resorption activity. During menopause, decreased estrogen levels lead to increased expression of Dkk1 and SOST, which inhibit the Wnt signaling pathway and disrupt bone metabolism (21). Inhibition of the Wnt/β-catenin signaling pathway results in reducing OPG expression and enhancing RANKL expression, which leads to a low OPG/RANKL ratio, elevating osteoclast activity and increasing bone resorption (54, 55).

The progression of osteoporosis during menopause is further aggravated by low mechanical loading on the bone, a decrease in osteocytes mechanosensitivity, which causes dysregulation of osteoblasts-osteoclasts interaction and leads to bone loss (42, 76).

The cellular response of osteocytes to mechanical stimulation is crucial for skeletal viability, and mechanosensitive ion channels Piezo1 are essential force sensors involved in bone development and in the regulation of osteoblast differentiation and function (39–41). Moreover, Piezo1 channels exist on the cell surface of osteocytes, osteoblasts, bone marrow mesenchymal stem cells and chondrocytes (41). The mechanical stain within the bone tissue during WBV or relate processes such as changes in intralacunar pressure or interstitial fluid flow, osteogenic processes are activated (48).

Osteocytes are stimulated by the physical WBV factors and represent the primary component of the skeleton responsible for maintaining the bone tissue environment and regulating osteoblasts and osteoclasts during the bone remodeling process (35). Activation of osteocytes suppresses the inhibitors of bone formation, such as sclerostin, while simultaneously, significantly increases RANKL expression (77). Sclerostin (encoded by the Sost gene), is a protein secreted primarily by osteocytes and an antagonist of the Wnt/β-catenin signaling pathway, inhibiting bone growth (78).

In our study postmenopausal women with osteoporosis demonstrated a significant increase in osteocalcin levels following WBV intervention compared with the control group (p <0.01). As is well known, osteocalcin acts as a versatile endocrine mediator at the interface of bone physiology and the systemic metabolic regulation (70). The osteocalcin difference between the EG and CG groups was p <0.01.

It can be assumed that WBV-induced physical factors stimulate the key mechanisms of bone formation involving osteocytes and osteoblasts, increase the OPG/RANKL ratio, and thereby suppresses bone resorption. Therefore, in our study it is evidenced by the increase in femoral BMD by 1.56% (p < 0.05).

Our conclusion regarding the activation of intercellular interactions between osteoblasts, osteoclasts and osteocytes under the influence of physical WBV factors is supported by the reported findings (62). According to their review, WBV activates osteoblast differentiation pathways (e.g., Wnt/β-catenin signaling pathway), increases the level of osteogenic markers (e.g., Runx2, BMP-2, OPG), and suppresses osteoclast activity (62).

We found that the osteogenic effect of WBV is closely associated with changes in biomarkers of innate and humoral immunity in postmenopausal women with osteoporosis (**Table 3**–**6**). Thus, we support the point of view that WBV in postmenopausal osteoporosis restores MBD through the interaction of immune cells and bone metabolism (60).

Our study found that the levels of the bone resorption marker β-CrossLaps in the blood serum of postmenopausal women with osteoporosis did not differ statistically between the three WBV program stages. Therefore, the function of the osteoclastic lineage does not dominate over the osteoblastic lineage under the WBV conditions in EG women.

### 4.1 The innate and adaptive immunity during the 6-months WBV program

It is widely recognized that the brain is tuned to detect immune signals, while neurotransmitters, in turn, have an immunostimulatory or immunosuppressive effects on both central and peripheral immune cells. This interaction is mediated by multiple immune pathways, including the endocrine one, and the effects of neurotransmitters vary depending on the receptor profile, signaling targets, and immune activation in the brain (79).

Menopause is characterized by hormonal changes and aging-related changes associated with immunological involution. Therefore, menopausal immune changes cannot be explained solely by estrogen deficiency. However, the literature lacks detailed description of specific age- and sex-related changes in the immune system (80). Moreover, earlier studies reported that during the immunological changes in the postmenopausal period, the total number of lymphocytes, particularly B cells and CD4+ T cells, tends to decline (81).

Recent data also indicate age-related involution of the cellular composition in leukocyte formula among pre- and menopausal women (82). Peripheral WBC is responsible for identifying and eliminating pathogens, clearing damaged cells and regulating immune responses. Overall, it was shown that for neutrophils, lymphocytes and monocytes, there was an interaction effect between age and specific time-depended segments across the menopause, with distinct trajectories for these three TWBC parameters, with decreasing trends and age-related interactions (83).

In our study, both the experimental (EG) and control group (CG) demonstrated a decrease in the percentage of neutrophils and monocytes within the TWBC structure in the peripheral blood during the 6-months WBV program (p < 0.05). In contrast, the percentage of lymphocytes within the WBC structure increased in both groups during the 3–6 month intervention period (p < 0.05). However, the magnitude of lymphocytes in the TWBC structure was significantly higher in the EG compared with the CG (p <0.05) during this period. This is a key indicator of immune system activation under the influence of 6-months WBV in postmenopausal women with osteoporosis.

As shown in **Table 3**, the number of cells involved in innate immunity changed differentially across the intervention. After 6 months of WBV, the number of neutrophils per milliliter of blood and their percentage in the leukocyte formula significantly decreased (p <0.05). A similar reduction was observed in the number of monocytes per milliliter of blood and their percentage in the leukocyte formula (p <0.05). Eosinophils and Basophils in TWBC showed multidirectional changes during the same period of the WBV intervention (**Table 3**).

In our study, at the month 3 of the WBV program, women in the EG demonstrated a significant increase in CD3+t (p <0.05) as a key indicator measuring the state of cellular immunity, since such lymphocytes are responsible for maintaining antigen homeostasis in the body. A similar effect was recently shown in a group of 16 men in traditional high-intensity exercise (84), whereas it was absent under moderate exercise. By month 6 of the WBV program, this indicator was lower in the EG than in the CG (p < 0.05) (**Table 4**).

These are important findings indicating that WBV provides enhanced functional responses to high-intensity activation of the integrative proprioceptive system in postmenopausal women with osteoporosis. Moreover, the adaptive immune response involving T-helpers (CD3+CD4+) decreases in the EG (p <0.05) at month 3 of the WBV program and is practically no different from the baseline after 6 months of WBV. T-lymphocytes, (CD3+ and CD8+ cells) are also considered a component of the adaptive immune response, assessing the immune system’s readiness to maintain overall health via immunosurveillance and immunoediting of the immune response (85).

In our study CD3+CD8+t cells count increased in the EG after 3 months of WBV, indicating an active immune response to high-intensity physical stimulation by WBV factors. Campbell et al. (86) previously in a randomized controlled trial investigated the impact of 12-months aerobic training exercise on immune function *in vitro* of 115 overweight/obese and sedentary postmenopausal women and reported no difference in NKCC activity between groups. It should be emphasized that WBV induces the highest activation of the integrative proprioceptive system and the osteogenic response than traditional exercise.

Numerous studies have examined the acute effects of exercise on circulating NK cells (CD3− CD16+ CD56+) due to their accessibility and significant changes in response to physical exercise (87). Moreover, the activity of NK cells during physical exercise also depends on the levels of hormones and cytokines in the blood (88).

In our study, by month 3 of the WBV program with high-intensity stimulation of the proprioceptive system, women in the EG exhibited decreased number of lymphocytes CD3+CD4+; CD-CD8+; CD3-CD3+CD16+ and the CD4+/CD8+ ratio (**Table 4**). By month 6, an increase in the number of NK cells was observed among lymphocyte subpopulations (CD3-CD20+) in EG women.

Finally, after 6 months of the WBV program, in EG women, serum levels of proinflammatory cytokines (IL-8, NFTα), as well as immunoglobulins (A, M, G) and the subpopulation composition of blood lymphocytes have their own characteristics in relation to the measurement periods (3 and 6 months) (**Table 6**). The activation of the immune system in postmenopausal women with osteoporosis under the influence of the 6-months WBV program is evidenced by the late marker CD3+DR+ (**Table 5**). The maximum concentration of IL-8 in the blood serum was detected after 3 months of WBV (**Table 6**), representing an increase from baseline “Before” (+42.8%), followed an increase at month 6 (+20.76%).

From the blood cell pool, IL-8 is produced by neutrophils, monocytes, T lymphocytes, for example, in response to inflammatory stimuli such as NFT-α (89), and, therefore, under the WBV influence, many physiological processes are physiologically initiated, including neutrophil recruitment and activation, as well as activation of other immune cells, including T cells and B cells (90). Increase in serum NFTα concentrations were observed after the month 3 of WBV (**Table 6**) that promotes the activation of the immune system to regulate immune responses in EG women during the WBV program, as can be expected from the literature (91). Furthermore, after 6-months WBV program implementation, NFTα levels significantly decreased below the NFTα levels observed in the “Before” stage of WBV.

### 4.2 The endocrine pathway of the brain-immune system relationship during WBV

It is well established that the innate immune system consists of a range of immune cell types, including monocytes, macrophages, dendritic cells, mast cells, neutrophils, eosinophils, basophils, natural killer cells, and innate lymphoid cells (92). The adaptive immune system consists of two main cell types (T-lymphocytes and B-lymphocytes), capable of generating antigen-specific responses against pathogens (93). Importantly, hormone receptors are expressed on the membrane of immune cells of both innate and adaptive immune systems (94). In addition to androgen receptor expression in mature immune cells, receptors are also expressed in hematopoietic stem cells, common myeloid progenitors, and common lymphoid progenitors within the bone marrow (95). Immunohistochemical analysis further demonstrates that all neutrophil lineages - from precursors to mature neutrophils – also express a cytoplasmic form of the androgen receptors in humans (96, 97). It is well known that the hormonal and non-hormonal biomarker responses to WBV are associated with increased serum concentrations of testosterone, growth hormone, epinephrine, norepinephrine, cortisol, as well as a number of cytokines (sclerostin adiponectin, irisin) and IGF-1 (67).

We propose that the mechanism initiating immune responses to the WBV physical factors involves the endocrine pathway, which mediates neuroimmune interactions. Furthermore, we assume that the key mechanism for increasing serum hormone concentrations in response to WBV is due to the integrative function of the proprioceptive sensory system (6) via a hypothetical proprioceptive-hypothalamic axis. This physiological mechanism requires experimental verification. The basis for this hypothesis lies in data on the physiological role of the proprioceptive-trunk loop, which modulates somatosensory-sympathetic activity via GABAergic interneurons n. tractus solitarius (98–100). Research by Pyatin and colleagues at the level of pons’ A5 area identified multisensory sympathoactivating neurons and proprioceptive interneurons (4). Activation of proprioceptive neurons in pons’ area A5 caused inhibition of the tonic electrical activity of multisensory sympathoactivating neurons and corresponding modulation of the regulatory effects of the sympathetic nervous system (101, 102). Based on these studies, we suggest that neural mechanisms of modulation of the somato-autonomous functions at the level of brainstem centers can be carried out through *the proprioceptive-brainstem axis.*

We assume that the hormonal responses to WBV are mediated through *the proprioceptive-hypothalamic axis*. As described in the literature, WBV-induced secretion of growth hormone (73, 74), testosterone (67, 73), cortisol (74), epinephrine and norepinephrine (75) can be modulated by ascending signals of the integrative proprioceptive system (6) via this axis. We make the fundamental assumption that these two axes represent the basis of modulating interactions between muscle-osteogenic signals on the one hand, and brainstem and hypothalamic structures on the other.

Serum concentrations of sclerostin, adiponectin, irisin, and IGF-1 reflect the activation of target tissue metabolism during WBV (103, 104). Peripheral mechanisms of integrative proprioceptive system activation during WBV determine non-hormonal biomarkers at the skeletal muscle level through the excretion of the myokine irisin, which regulates fat, muscle, and bone metabolism (103). The cytokine adiponectin is implicated as a mediator of the fat-bone relationship, controlling the negative relation between blood adiponectin concentrations and BMD. So, adipoR1 and R2 are expressed in human primary osteoblasts and in bone marrow macrophages and stimulate osteoclast differentiation (105). Plasma sclerostin level may be used as an indicator to evaluate the bone response to mechanical loading and is expressed by osteocytes (104). Finally, insulin-like growth factor 1 (IGF1) is a peptide growth factor with important functions in multiple aspects of growth, development and metabolism (106).

Thus, the innate and adaptive immune responses observed in women of the EG under WBV are mediated by the endocrine pathway as an interface between the brain and the immune system, and osteogenic structures. This conceptual hypothesis is illustrated in **Figure 1**.

The significance of the endocrine pathway in mediating the brain-immune system axis interactions is supported by multiple studies revealing the nature of hormonal modulation of the brain-immune system axis. According to these data, hormone receptors are expressed on the membrane of peripheral blood cells, and serum levels of these receptors increase by the physical factors during the WBV intervention. These data reflect a complex molecular interface that mediates the endocrine and immune systems interaction. For instance, this is the control of progesterone by membrane progesterone receptors (mPR) expression in peripheral blood leukocytes of reproductive-aged women (107). Immune cell populations from both the innate and adaptive immune systems express androgen receptor (AR) through direct and indirect AR signaling (95, 108). β1- and β2-Adrenergic Receptors are found on PBMCs, T cells, monocytes, and NK cells (109).

Membrane estrogen receptors ERβ and ERα have been suggested by a number of observations (110). Growth hormone and growth hormone-releasing receptors are expressed on various blood cells, including lymphocytes (T and B cells), neutrophils, and, thus, indicating that growth hormone can modulate immune responses such as T and B cell proliferation and cytokine production (111). Glucocorticoids, in addition to the effect of activating the cytosolic glucocorticoid receptor (cGR), which translocate to the nucleus and regulates gene expression (4), exhibit rapid nongenomic GC-activation effects via membrane-bound GR (mGR) (112). Finally, expression of mineralocorticoid receptors has also been shown in most PBMC subsets (113, 114), including T and B lymphocytes, expanding our understanding of the modulatory role of the endocrine system in immune functions.

### 4.3 Osteogenic function of the endocrine pathway in WBV

Continuing our working hypothesis, we argue that the endocrine pathway modulates not only the interaction between the brain and the immune system, but also regulates the osteogenic functions of osteoblasts, osteoclasts and osteocytes during WBV in postmenopausal women with osteoporosis. This feedback of growth hormone, IGF-1, testosterone, epinephrine, norepinephrine and cortisol in WBV is mediated through the membrane receptor mechanisms and osteocyte, osteoblast, and osteoclast pathways.

According to the literature, it is known that under the influence of IGF-1, the level of osteoclastic activity markers decreases with the normalization of the calcitonin receptor gene (CTR) (115). Moreover, modulation of bone repair and regeneration with the IGF-1 participation, along with other hormones and regulatory cytokines, is carried out through Wnt/β-catenin, bone morphogenetic proteins (BMP) and fibroblast growth factor (FGF) signaling pathways (116).

Our study found that the elevated level of the IGF-1 biomarker can cause the activation of the osteoblastic lineage under the influence of WBV physical factors in postmenopausal women with osteoporosis.

According to literature data the glucocorticoids reduce cell replication of osteoblastic lineage and osteoblast function through direct effects on the osteoclast lineage and indirect effects on osteoblasts and T cells mediated by cytokines such as RANKL, osteoprotegerin, and TNF-α (117–119). It is also known that after exposure to WBV physical factors, cortisol levels in the blood plasma of the test subjects decrease (74). This path can also contribute to the activity of the osteoblastic lineage in our study.

Catecholamines activate β2-ARs on the membranes of osteoblasts and osteoclasts, which has various consequences for bone homeostasis. Stimulation of β-ARs, through various direct or indirect mechanisms, promotes osteoclast activity and reduces bone mass. So, the stimulation of β-ARs inhibits osteoblast proliferation and bone issue formation, increasing bone resorption (120, 121). However, in the absence of dynamics in the serum levels of the bone resorption marker β-CrossLaps in EG between the three stages of the WBV program the role of the catecholamines rests discuss.

Our study demonstrated an increase in blood osteocalcin levels during the WBV 6-month sessions in postmenopausal women with osteoporosis. Osteocalcin (OCN), as shown in several studies, is secreted by osteoblasts and, thus, is a key marker of bone formation and metabolism, functioning as an endocrine hormone (122, 123).

Finally, regarding the mechanisms of growth hormone action on osteogenesis, a stimulatory effect on OPG production has been demonstrated in tissue culture (124). This suggests that growth hormone directly modulates the interaction between osteoblasts and osteoclasts, thereby promoting bone remodeling.

Therefore, it can be concluded that the osteogenic function of the endocrine pathway is involved in modulating the homeostasis of intercellular interactions in bone tissue, which is simultaneously under local mechanosensory modulation of the osteogenic functions of osteoblasts, osteoclasts and osteocytes, and the balance of interactions between them. In addition, **Figure 1** shows the summary analysis of the putative role of the endocrine pathway in modulating the physiological mechanisms of bone remodeling during the WBV physical factors on the musculoskeletal system in postmenopausal women with osteoporosis. First of all, the endocrine pathway is modulated by the integrative proprioceptive system. The consequence of this process is hormonal modulation of the molecular mechanisms of innate and adaptive immunity. Simultaneously, the endocrine pathway has feedback with osteoblasts and osteoclasts of osteogenesis via a hormonal loop. According to our working hypothesis, the interaction between these mechanisms represents the basis for non-pharmacological functional remodeling of BMD in postmenopausal women with osteoporosis during WBV.

## 5 Conclusion

In our study, the longitudinal data obtained on BMD, osteocalcin, immune subpopulations and cytokines under the WBV influence reveal a complex network of physiological relationships that modulate osteogenesis in postmenopausal women and restore BMD. It is well known, the network of physiological links connecting bone metabolism and branches of the immune system in relation to physical exercise—and even more so to WBV to date - has been described only in a limited manner. As a result of our research and analysis of the literature on the equally complex links between WBV and endocrine system responses, we have formulated, for the first time, a hypothesis regarding brain-immune interaction during the activation of osteogenesis in postmenopausal women with osteoporosis. In this interaction an integrative proprioceptive-endocrine pathway linking the brain, the immune system and bone remodeling during WBV can be considered as the interface for cross-system communication. Our concept of an integrative proprioceptive endocrine pathway as an interface between the brain’s proprioceptive integrative system and the immune system is supported by literature data on immune system cells possessing membrane receptors for hormones secreted into the blood plasma during WBV. This suggests that the integrative proprioceptive-endocrine pathway can serve as an intersystem interface. In turn, cells of the osteogenic pool (osteoblasts, osteoclasts and osteocytes), according to the literature, are subject to the modulatory influence of hormones of endocrine system and the immune regulation.

In conclusion, it can be said that the osteogenic potential of WBV is realised at the several interacting modulation levels of BMD remodeling: the musculoskeletal level with the participation of the receptor link of the integrative proprioceptive system and the mechanosensory function of bone osteocytes; the endocrine pathway of interaction between the integrative proprioceptive system and the brain; the endocrine pathway of interaction between the brain and the immune system; the endocrine pathway of interaction between the brain, the osteoblastic and osteoclastic lineages of bones and the stimulation of osteogenesis (**Figure 1**). The application of WBV within the framework of our hypothesis, which links osteoimmunology to the broader systemic regulation of osteogenesis, is of clinical significance given the need for accessible methods of functional treatment for an ageing population.

## 6 Limitations

The study is limited by the lack of our original data on serum hormone concentrations in postmenopausal women with osteoporosis during the 6-months WBV intervention. Current literature also provides only limited evidence regarding membrane expression of hormone receptors on the immune system cells and bone-forming cells of bone tissue. The study conducted only three-stage monitoring of the bone issue remodeling biomarkers, as well as the biomarkers of innate and adaptive immunity during the 6-months WBV program. In our study, heart rate variability parameters were not recorded in EG women to draw conclusion about the degree of sympathetic nervous system tone during WBV in postmenopausal women. Further studies on the role of the endocrine pathway in BMD remodeling in postmenopausal women with osteoporosis are needed, considering the new ideas of our working hypothesis.

## Data Availability

The raw data supporting the conclusions of this article will be made available by the authors, without undue reservation.
